# Benzene-1,3,5-tricarb­oxy­lic acid–5-(pyridin-1-ium-3-yl)-5*H*-1,2,3,4-tetra­zol-5-ide (1/1)

**DOI:** 10.1107/S1600536811021490

**Published:** 2011-06-11

**Authors:** Gao-Xiang Meng, Hao Ding, Ya-Min Feng, Jian-Hui Zhu, He-Lin Yang

**Affiliations:** a Department of Physics, Central China Normal University, Wuhan 430079, People’s Republic of China

## Abstract

The asymmetric unit of the title compound, C_6_H_5_N_5_·C_9_H_6_O_6_, comprises a full mol­ecule each of neutral trimesic acid (tma) and zwitterionic 5-(pyridin-1-ium-3-yl)-5*H*-1,2,3,4-tetra­zol-5-ide (ptz). The components are linked into a two-dimensional layer by a combination of O—H⋯O, O—H⋯N, N—H⋯O and N—H⋯N hydrogen bonds parallel to the (10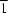) plane. Layers comprising alternating rows of tma and ptz are linked into a three-dimensional network by C—H⋯O and π–π inter­actions between tma and tetra­zolate rings [centroid–centroid distance = 3.763 (2) Å], and between pyridinium and tetra­zolate rings [centroid–centroid distance = 3.745 (2) Å].

## Related literature

For crystal engineering studies involving the components of the title compound, see: Lin *et al.* (2005 ▶); Luo *et al.* (2005 ▶); Yang *et al.* (2011 ▶).
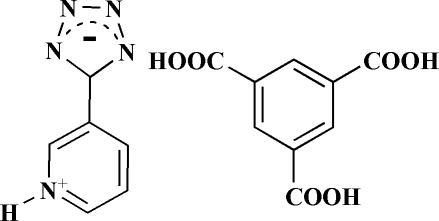


## Experimental

### 

#### Crystal data

C_6_H_5_N_5_·C_9_H_6_O_6_
*M* *_r_* = 357.29
Triclinic, 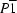

*a* = 7.6596 (8) Å
*b* = 8.7374 (9) Å
*c* = 11.3931 (11) Å
α = 94.336 (2)°
β = 95.584 (1)°
γ = 98.465 (2)°
*V* = 747.46 (13) Å^3^
*Z* = 2
Mo *K*α radiation
μ = 0.13 mm^−1^
*T* = 298 K
0.16 × 0.12 × 0.10 mm

#### Data collection

Bruker SMART APEX CCD area-detector diffractometer
Absorption correction: multi-scan (*SADABS*; Sheldrick, 1997 ▶) *T*_min_ = 0.970, *T*_max_ = 0.988
7554 measured reflections
2770 independent reflections
2438 reflections with *I* > 2σ(*I*)
*R*_int_ = 0.028

#### Refinement

*R*[*F* ^2^ > 2σ(*F* ^2^)] = 0.068
*wR*(*F* ^2^) = 0.139
*S* = 1.24
2770 reflections
248 parameters
H atoms treated by a mixture of independent and constrained refinement
Δρ_max_ = 0.24 e Å^−3^
Δρ_min_ = −0.29 e Å^−3^

### 

Data collection: *SMART* (Bruker, 2001 ▶); cell refinement: *SAINT-Plus* (Bruker, 2001 ▶); data reduction: *SAINT-Plus*; program(s) used to solve structure: *SHELXS97* (Sheldrick, 2008 ▶); program(s) used to refine structure: *SHELXL97* (Sheldrick, 2008 ▶); molecular graphics: *PLATON* (Spek, 2009 ▶); software used to prepare material for publication: *PLATON*.

## Supplementary Material

Crystal structure: contains datablock(s) global, I. DOI: 10.1107/S1600536811021490/tk2753sup1.cif

Structure factors: contains datablock(s) I. DOI: 10.1107/S1600536811021490/tk2753Isup2.hkl

Supplementary material file. DOI: 10.1107/S1600536811021490/tk2753Isup3.cml

Additional supplementary materials:  crystallographic information; 3D view; checkCIF report

## Figures and Tables

**Table 1:** Hydrogen-bond geometry (Å, °)

*D*—H⋯*A*	*D*—H	H⋯*A*	*D*⋯*A*	*D*—H⋯*A*
N1—H1⋯N4^i^	0.88 (4)	2.25 (4)	2.981 (3)	141 (3)
N1—H1⋯O3	0.88 (4)	2.49 (3)	2.958 (3)	114 (3)
O1—H1*A*⋯O5^ii^	0.83 (5)	1.88 (5)	2.694 (3)	165 (5)
O4—H4*A*⋯N3^i^	0.92 (4)	1.73 (4)	2.635 (3)	168 (4)
O6—H6*A*⋯N4^iii^	0.98 (4)	2.57 (4)	3.325 (3)	134 (3)
O6—H6*A*⋯N5^iii^	0.98 (4)	1.70 (4)	2.647 (3)	163 (3)
C3—H3⋯O6^iv^	0.93	2.41	3.230 (3)	147
C4—H4⋯O2^v^	0.93	2.54	3.418 (4)	157

Symmetry codes: (i) 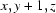; (ii) 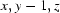; (iii) 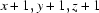; (iv) 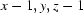; (v) 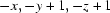.

**Fig. 1.**
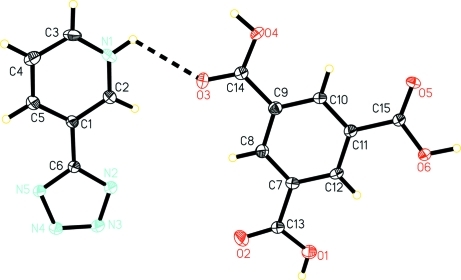
The molecular structures of the constituents of (I), showing the atom-numbering scheme. Displacement ellipsoids are drawn at the 50% probability level and H atoms are shown as small spheres of arbitrary radii. The hydrogen bond is shown as a dashed line.

**Fig. 2.**
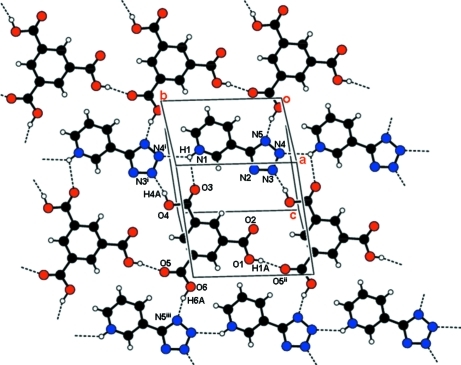
Part of the crystal structure of (I), showing the formation of the two-dimensional layer parallel to the (101) plane stabilised by a combination of O—H···O, O—H···N, N—H···O and N—H···N hydrogen bonds. Symmetry codes: i) x, 1 + y, z; ii) x, y - 1, z; iii) 1 + x, 1 + y, 1 + z.

**Fig. 3.**
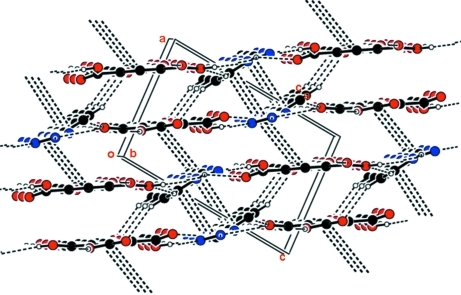
Part of the crystal structure of (I), showing the formation of the three-dimensional network by a combination of hydrogen bonds (see Fig. 2), C—H···O and π–π interactions (dashed lines). Hydrogen atoms not involved in significant intermolecular contacts have been omitted for clarity.

## supplementary crystallographic information

### Comment

Recently, pyridyl-tetrazole ligands have been used in the construction of metal-organic complexes (Yang, *et al.*, 2011; Lin *et al.*, 2005; Luo, *et al.*, 2005). In the reaction of trimesic acid (tma), 5-(3-pyridyl)tetrazolate (ptz) and CdSO_4_ under hydrothermal conditions, we have unexpectedly obtained the title compound, (I), and determined its crystal structure.

In (I), the asymmetric unit comprises a neutral trimesic acid (tma) molecule and a 5-(3-pyridinium)tetrazolate (ptz) zwitterion (Fig. 1). In tma, the three carboxyl hydrogen atoms were readily found from the difference maps. This, coupled with the disparity in the C—O bond distances, confirms its neutral composition. The three carboxyl groups are twisted away from the central benzene plane forming dihedral angles of 6.59 (17) °, 16.12 (16) ° and 7.10 (16) ° between the groups containing the O1, O3 and O5 atoms, respectively. In the ptz zwitterion, the tetrazole ring is twisted away from the adjacent benzene ring with the dihedral angle between them being 16.38 (16) °.

In the crystal packing of (I), the component species are linked into a two-dimensional array running parallel to the (101) plane by a combination of O—H···O, O—H···N, N—H···O and N—H···N hydrogen bonds (Fig. 2 and Table 2). The layer can be analysed in terms of two sub-structures. Firstly, by the O1—H1A···O5 (*x*, *y* - 1, *z*) and N—H1···N4 (*x*, 1 + *y*, *z*) hydrogen bonds which link tma and 3-ptz ions into one-dimensional chains along the *c* axis (Fig. 2). Secondly, adjacent chains are joined together by the N1—H1···O3, O4—H4A···N3 (*x*, 1 + *y*, *z*) and O6—H6A···N5 (1 + *x*, 1 + *y*, 1 + *z*) hydrogen bonds, resulting in the formation of a two-dimensional layer parallel to the (101) plane (Fig. 2). Further analysis indicates that these adjacent layers are linked into the three-dimensional network by a combination of a C4—H4···O2 (-*x*,1 - *y*,1 - *z*) contacts and two π–π interactions (Fig. 3), one occurs between symmetry-related tma and tetrazolate rings [centroid distance: 3.763 (2) Å, dihedral angle: 11.10 (15) °, symmetry code: 1 - *x*, 1 - *y*, 1 - *z*], and the other occurs between symmetry-related pyridinium and tetrazolate rings [centroid distance: 3.745 (2) Å, dihedral angle: 16.38 (16)°, symmetry code: -*x*, 1 - *y*, 1 - *z*].

### Experimental

All the reagents and solvents were used as obtained without further purification. Equivalent molar amounts of trimesic acid, 5-(3-pyridyl)tetrazole and CdSO_4_ were reacted under hydrothermal conditions. The mixture was sealed in a 23 cm^3^ Teflon-lined stainless steel container. The temperature was kept at 433 K for 4 days and cooled to room temperature at the rate of 5 K /h. Colourless crystals of (I) were obtained and separated manually.

### Refinement

H atoms bonded to C atoms were positioned geometrically with C–H = 0.93 Å and refined in a riding mode with *U*_iso_(H) = 1.2*U*_eq_(C). H atoms bonded to O and N were found in difference maps. Their positions were refined freely (see Table 1) with *U*_iso_(H) = 1.2 (N) or 1.5 (O) times *U*_eq_(parent atom).

### Crystal data

**Table:**

C_6_H_5_N_5_·C_9_H_6_O_6_	*Z* = 2
*M**_r_* = 357.29	*F*(000) = 368
Triclinic, *P*1	*D*_x_ = 1.587 Mg m^−^^3^
Hall symbol: -p 1	Mo *K*α radiation, λ = 0.71073 Å
*a* = 7.6596 (8) Å	Cell parameters from 2432 reflections
*b* = 8.7374 (9) Å	θ = 2.4–28.3°
*c* = 11.3931 (11) Å	µ = 0.13 mm^−^^1^
α = 94.336 (2)°	*T* = 298 K
β = 95.584 (1)°	Block, colourless
γ = 98.465 (2)°	0.16 × 0.12 × 0.10 mm
*V* = 747.46 (13) Å^3^	

### Data collection

**Table:**

Bruker SMART APEX CCD area-detector diffractometer	2770 independent reflections
Radiation source: fine focus sealed Siemens Mo tube	2438 reflections with *I* > 2σ(*I*)
graphite	*R*_int_ = 0.028
0.3° wide ω exposures scans	θ_max_ = 25.5°, θ_min_ = 2.4°
Absorption correction: multi-scan (*SADABS*; Sheldrick, 1997)	*h* = −9→9
*T*_min_ = 0.970, *T*_max_ = 0.988	*k* = −10→10
7554 measured reflections	*l* = −13→13

### Refinement

**Table:**

Refinement on *F*^2^	Secondary atom site location: difference Fourier map
Least-squares matrix: full	Hydrogen site location: inferred from neighbouring sites
*R*[*F*^2^ > 2σ(*F*^2^)] = 0.068	H atoms treated by a mixture of independent and constrained refinement
*wR*(*F*^2^) = 0.139	*w* = 1/[σ^2^(*F*_o_^2^) + (0.041*P*)^2^ + 0.5688*P*] where *P* = (*F*_o_^2^ + 2*F*_c_^2^)/3
*S* = 1.24	(Δ/σ)_max_ = 0.005
2770 reflections	Δρ_max_ = 0.24 e Å^−^^3^
248 parameters	Δρ_min_ = −0.29 e Å^−^^3^
0 restraints	Extinction correction: *SHELXL97* (Sheldrick, 2008), Fc^*^=kFc[1+0.001xFc^2^λ^3^/sin(2θ)]^-1/4^
Primary atom site location: structure-invariant direct methods	Extinction coefficient: 0

### Special details

**Table:**

Geometry. All e.s.d.'s (except the e.s.d. in the dihedral angle between two l.s. planes) are estimated using the full covariance matrix. The cell e.s.d.'s are taken into account individually in the estimation of e.s.d.'s in distances, angles and torsion angles; correlations between e.s.d.'s in cell parameters are only used when they are defined by crystal symmetry. An approximate (isotropic) treatment of cell e.s.d.'s is used for estimating e.s.d.'s involving l.s. planes.
Refinement. Refinement of *F*^2^ against ALL reflections. The weighted *R*-factor *wR* and goodness of fit *S* are based on *F*^2^, conventional *R*-factors *R* are based on *F*, with *F* set to zero for negative *F*^2^. The threshold expression of *F*^2^ > σ(*F*^2^) is used only for calculating *R*-factors(gt) *etc*. and is not relevant to the choice of reflections for refinement. *R*-factors based on *F*^2^ are statistically about twice as large as those based on *F*, and *R*- factors based on ALL data will be even larger.

### Fractional atomic coordinates and isotropic or equivalent isotropic displacement parameters (Å^2^)

**Table:**

	*x*	*y*	*z*	*U*_iso_*/*U*_eq_	
C1	0.0853 (4)	0.4773 (3)	0.3543 (2)	0.0287 (6)	
C2	0.1850 (4)	0.6185 (3)	0.3963 (3)	0.0380 (7)	
H2	0.2941	0.6226	0.4410	0.046*	
C3	−0.0294 (5)	0.7514 (3)	0.3090 (3)	0.0442 (8)	
H3	−0.0663	0.8458	0.2951	0.053*	
C4	−0.1328 (4)	0.6155 (4)	0.2643 (3)	0.0438 (8)	
H4	−0.2406	0.6158	0.2190	0.053*	
C5	−0.0751 (4)	0.4771 (3)	0.2873 (3)	0.0378 (7)	
H5	−0.1445	0.3833	0.2575	0.045*	
C6	0.1461 (4)	0.3330 (3)	0.3868 (2)	0.0271 (6)	
C7	0.6435 (4)	0.6942 (3)	0.8877 (2)	0.0287 (6)	
C8	0.5465 (4)	0.7376 (3)	0.7906 (3)	0.0306 (6)	
H8	0.4858	0.6622	0.7331	0.037*	
C9	0.5391 (4)	0.8940 (3)	0.7785 (2)	0.0283 (6)	
C10	0.6274 (4)	1.0065 (3)	0.8654 (2)	0.0287 (6)	
H10	0.6234	1.1111	0.8571	0.034*	
C11	0.7210 (4)	0.9634 (3)	0.9639 (2)	0.0277 (6)	
C12	0.7303 (4)	0.8069 (3)	0.9750 (2)	0.0301 (6)	
H12	0.7947	0.7779	1.0409	0.036*	
C13	0.6573 (4)	0.5263 (3)	0.8965 (2)	0.0324 (7)	
C14	0.4369 (4)	0.9377 (3)	0.6711 (2)	0.0313 (7)	
C15	0.8122 (4)	1.0852 (3)	1.0584 (2)	0.0295 (6)	
N1	0.1243 (4)	0.7486 (3)	0.3726 (3)	0.0456 (7)	
H1	0.183 (5)	0.841 (4)	0.401 (3)	0.055*	
N2	0.2717 (3)	0.3285 (3)	0.4749 (2)	0.0366 (6)	
N3	0.2783 (3)	0.1772 (3)	0.4792 (2)	0.0364 (6)	
N4	0.1635 (3)	0.0944 (3)	0.3974 (2)	0.0342 (6)	
N5	0.0770 (3)	0.1911 (2)	0.3364 (2)	0.0316 (6)	
O1	0.7675 (4)	0.5045 (3)	0.9864 (2)	0.0643 (8)	
H1A	0.779 (6)	0.412 (5)	0.993 (4)	0.096*	
O2	0.5762 (4)	0.4234 (3)	0.8289 (2)	0.0646 (8)	
O3	0.3320 (3)	0.8463 (2)	0.60580 (18)	0.0423 (6)	
O4	0.4766 (3)	1.0861 (2)	0.6547 (2)	0.0474 (6)	
H4A	0.406 (5)	1.104 (4)	0.589 (4)	0.071*	
O5	0.8228 (3)	1.2228 (2)	1.04881 (19)	0.0470 (6)	
O6	0.8802 (3)	1.0282 (2)	1.15213 (18)	0.0409 (6)	
H6A	0.944 (5)	1.106 (4)	1.214 (3)	0.061*	

### Atomic displacement parameters (Å^2^)

**Table:**

	*U*^11^	*U*^22^	*U*^33^	*U*^12^	*U*^13^	*U*^23^
C1	0.0376 (16)	0.0218 (14)	0.0248 (14)	0.0026 (12)	−0.0011 (12)	0.0003 (11)
C2	0.0460 (19)	0.0257 (15)	0.0389 (17)	0.0041 (13)	−0.0080 (14)	0.0004 (13)
C3	0.064 (2)	0.0238 (16)	0.0460 (19)	0.0112 (15)	0.0001 (17)	0.0118 (14)
C4	0.051 (2)	0.0391 (18)	0.0425 (19)	0.0149 (15)	−0.0078 (15)	0.0090 (14)
C5	0.0473 (18)	0.0262 (15)	0.0363 (17)	0.0028 (13)	−0.0066 (14)	−0.0003 (12)
C6	0.0311 (15)	0.0237 (14)	0.0254 (14)	0.0029 (11)	−0.0004 (12)	0.0026 (11)
C7	0.0331 (15)	0.0231 (14)	0.0302 (15)	0.0049 (11)	0.0026 (12)	0.0055 (11)
C8	0.0295 (15)	0.0293 (15)	0.0315 (15)	0.0017 (12)	−0.0008 (12)	0.0047 (12)
C9	0.0288 (15)	0.0288 (14)	0.0285 (15)	0.0074 (12)	0.0039 (12)	0.0033 (12)
C10	0.0353 (16)	0.0227 (14)	0.0284 (15)	0.0068 (11)	0.0000 (12)	0.0056 (11)
C11	0.0301 (15)	0.0266 (14)	0.0267 (15)	0.0071 (11)	0.0005 (12)	0.0035 (11)
C12	0.0362 (16)	0.0271 (14)	0.0277 (15)	0.0085 (12)	−0.0034 (12)	0.0086 (11)
C13	0.0412 (17)	0.0260 (15)	0.0293 (16)	0.0048 (13)	−0.0011 (13)	0.0047 (12)
C14	0.0341 (16)	0.0288 (15)	0.0305 (16)	0.0064 (12)	−0.0008 (13)	0.0038 (12)
C15	0.0353 (16)	0.0260 (15)	0.0268 (15)	0.0077 (12)	−0.0034 (12)	0.0026 (11)
N1	0.0549 (18)	0.0200 (13)	0.0564 (18)	−0.0011 (12)	−0.0101 (14)	0.0019 (12)
N2	0.0457 (15)	0.0275 (13)	0.0339 (14)	0.0054 (11)	−0.0084 (12)	0.0032 (10)
N3	0.0462 (15)	0.0286 (13)	0.0332 (14)	0.0087 (11)	−0.0061 (12)	0.0039 (11)
N4	0.0411 (14)	0.0240 (12)	0.0374 (14)	0.0079 (11)	−0.0017 (11)	0.0039 (10)
N5	0.0410 (14)	0.0228 (12)	0.0294 (13)	0.0063 (10)	−0.0063 (11)	0.0030 (10)
O1	0.105 (2)	0.0261 (12)	0.0554 (16)	0.0204 (13)	−0.0349 (15)	0.0024 (11)
O2	0.0873 (19)	0.0271 (12)	0.0693 (17)	0.0070 (12)	−0.0330 (15)	−0.0036 (11)
O3	0.0476 (13)	0.0375 (12)	0.0355 (12)	0.0017 (10)	−0.0160 (10)	−0.0008 (9)
O4	0.0598 (15)	0.0344 (12)	0.0418 (13)	0.0045 (10)	−0.0263 (11)	0.0105 (10)
O5	0.0709 (16)	0.0194 (11)	0.0467 (14)	0.0101 (10)	−0.0186 (11)	0.0025 (9)
O6	0.0589 (14)	0.0245 (11)	0.0344 (12)	0.0056 (10)	−0.0194 (10)	0.0040 (9)

### Geometric parameters (Å, °)

**Table:**

C1—C2	1.377 (4)	C9—C14	1.493 (4)
C1—C5	1.382 (4)	C10—C11	1.379 (4)
C1—C6	1.468 (4)	C10—H10	0.9300
C2—N1	1.326 (4)	C11—C12	1.394 (4)
C2—H2	0.9300	C11—C15	1.497 (4)
C3—N1	1.325 (4)	C12—H12	0.9300
C3—C4	1.360 (4)	C13—O2	1.193 (3)
C3—H3	0.9300	C13—O1	1.307 (4)
C4—C5	1.382 (4)	C14—O3	1.201 (3)
C4—H4	0.9300	C14—O4	1.319 (3)
C5—H5	0.9300	C15—O5	1.207 (3)
C6—N2	1.328 (3)	C15—O6	1.307 (3)
C6—N5	1.337 (3)	N1—H1	0.88 (4)
C7—C8	1.381 (4)	N2—N3	1.335 (3)
C7—C12	1.390 (4)	N3—N4	1.310 (3)
C7—C13	1.496 (4)	N4—N5	1.341 (3)
C8—C9	1.392 (4)	O1—H1A	0.83 (5)
C8—H8	0.9300	O4—H4A	0.92 (4)
C9—C10	1.390 (4)	O6—H6A	0.98 (4)
			
C2—C1—C5	118.1 (3)	C11—C10—H10	120.0
C2—C1—C6	119.8 (3)	C9—C10—H10	120.0
C5—C1—C6	122.0 (2)	C10—C11—C12	119.9 (2)
N1—C2—C1	119.6 (3)	C10—C11—C15	119.7 (2)
N1—C2—H2	120.2	C12—C11—C15	120.3 (2)
C1—C2—H2	120.2	C7—C12—C11	120.1 (2)
N1—C3—C4	119.7 (3)	C7—C12—H12	120.0
N1—C3—H3	120.2	C11—C12—H12	120.0
C4—C3—H3	120.2	O2—C13—O1	123.6 (3)
C3—C4—C5	118.8 (3)	O2—C13—C7	123.7 (3)
C3—C4—H4	120.6	O1—C13—C7	112.6 (2)
C5—C4—H4	120.6	O3—C14—O4	123.9 (3)
C1—C5—C4	120.4 (3)	O3—C14—C9	123.2 (3)
C1—C5—H5	119.8	O4—C14—C9	112.9 (2)
C4—C5—H5	119.8	O5—C15—O6	123.2 (3)
N2—C6—N5	112.1 (2)	O5—C15—C11	123.3 (2)
N2—C6—C1	123.2 (2)	O6—C15—C11	113.5 (2)
N5—C6—C1	124.6 (2)	C3—N1—C2	123.4 (3)
C8—C7—C12	119.8 (2)	C3—N1—H1	115 (2)
C8—C7—C13	119.6 (2)	C2—N1—H1	122 (2)
C12—C7—C13	120.7 (2)	C6—N2—N3	104.0 (2)
C7—C8—C9	120.2 (3)	N4—N3—N2	110.7 (2)
C7—C8—H8	119.9	N3—N4—N5	108.6 (2)
C9—C8—H8	119.9	C6—N5—N4	104.6 (2)
C10—C9—C8	119.9 (3)	C13—O1—H1A	115 (3)
C10—C9—C14	121.1 (2)	C14—O4—H4A	107 (2)
C8—C9—C14	119.0 (2)	C15—O6—H6A	115 (2)
C11—C10—C9	120.1 (2)		
			
C5—C1—C2—N1	0.7 (4)	C8—C7—C13—O2	−6.8 (5)
C6—C1—C2—N1	−176.0 (3)	C12—C7—C13—O2	174.6 (3)
N1—C3—C4—C5	0.5 (5)	C8—C7—C13—O1	173.4 (3)
C2—C1—C5—C4	−0.4 (5)	C12—C7—C13—O1	−5.2 (4)
C6—C1—C5—C4	176.2 (3)	C10—C9—C14—O3	−165.2 (3)
C3—C4—C5—C1	−0.2 (5)	C8—C9—C14—O3	15.3 (4)
C2—C1—C6—N2	15.2 (4)	C10—C9—C14—O4	16.0 (4)
C5—C1—C6—N2	−161.3 (3)	C8—C9—C14—O4	−163.6 (3)
C2—C1—C6—N5	−168.4 (3)	C10—C11—C15—O5	−7.1 (4)
C5—C1—C6—N5	15.1 (4)	C12—C11—C15—O5	172.9 (3)
C12—C7—C8—C9	1.6 (4)	C10—C11—C15—O6	173.3 (3)
C13—C7—C8—C9	−177.0 (3)	C12—C11—C15—O6	−6.7 (4)
C7—C8—C9—C10	−1.0 (4)	C4—C3—N1—C2	−0.3 (5)
C7—C8—C9—C14	178.5 (3)	C1—C2—N1—C3	−0.4 (5)
C8—C9—C10—C11	−0.5 (4)	N5—C6—N2—N3	−0.6 (3)
C14—C9—C10—C11	180.0 (3)	C1—C6—N2—N3	176.2 (3)
C9—C10—C11—C12	1.4 (4)	C6—N2—N3—N4	0.4 (3)
C9—C10—C11—C15	−178.6 (3)	N2—N3—N4—N5	−0.1 (3)
C8—C7—C12—C11	−0.7 (4)	N2—C6—N5—N4	0.6 (3)
C13—C7—C12—C11	178.0 (3)	C1—C6—N5—N4	−176.2 (3)
C10—C11—C12—C7	−0.8 (4)	N3—N4—N5—C6	−0.3 (3)
C15—C11—C12—C7	179.2 (3)		

### Hydrogen-bond geometry (Å, °)

**Table:**

*D*—H···*A*	*D*—H	H···*A*	*D*···*A*	*D*—H···*A*
N1—H1···N4^i^	0.88 (4)	2.25 (4)	2.981 (3)	141 (3)
N1—H1···O3	0.88 (4)	2.49 (3)	2.958 (3)	114 (3)
O1—H1A···O5^ii^	0.83 (5)	1.88 (5)	2.694 (3)	165 (5)
O4—H4A···N3^i^	0.92 (4)	1.73 (4)	2.635 (3)	168 (4)
O6—H6A···N4^iii^	0.98 (4)	2.57 (4)	3.325 (3)	134 (3)
O6—H6A···N5^iii^	0.98 (4)	1.70 (4)	2.647 (3)	163 (3)
C3—H3···O6^iv^	0.93	2.41	3.230 (3)	147.
C4—H4···O2^v^	0.93	2.54	3.418 (4)	157.

Symmetry codes: (i) *x*, *y*+1, *z*; (ii) *x*, *y*−1, *z*; (iii) *x*+1, *y*+1, *z*+1; (iv) *x*−1, *y*, *z*−1; (v) −*x*, −*y*+1, −*z*+1.
